# ELISA-Based Measurement of Antibody Responses and PCR-Based Detection Profiles Can Distinguish between Active Infection and Early Clearance of *Borrelia burgdorferi*

**DOI:** 10.1155/2012/138069

**Published:** 2011-10-26

**Authors:** John J. Lazarus, Akisha L. McCarter, Kari Neifer-Sadhwani, R. Mark Wooten

**Affiliations:** Department of Medical Microbiology and Immunology, University of Toledo College of Medicine, Toledo, OH 43614, USA

## Abstract

*Borrelia burgdorferi* is a spirochetal bacterium that causes Lyme disease. These studies address whether current research methods using either ELISA to detect seroconversion to *B. burgdorferi* antigens or PCR quantification of bacterial DNA within tissues can accurately distinguish between a productive infection versus a *B. burgdorferi* exposure that is rapidly cleared by the innate responses. Mice receiving even minimal doses of live *B. burgdorferi* produced significantly more *B. burgdorferi*-specific IgM and IgG than groups receiving large inocula of heat-killed bacteria. Additionally, sera from mice injected with varied doses of killed *B. burgdorferi* recognized unique borrelial antigens compared to mice infected with live *B. burgdorferi*. Intradermal injection of killed *B. burgdorferi* resulted in rapid DNA clearance from skin, whereas DNA was consistently detected in skin inoculated with viable *B. burgdorferi*. These data indicate that both ELISA-based serological analyses and PCR-based methods of assessing *B. burgdorferi* infection clearly distinguish between an established infection with live bacteria and exposure to large numbers of bacteria that are promptly cleared by the innate responses.

## 1. Introduction

*Borrelia burgdorferi* (Bb) is a spirochetal bacterium that causes Lyme disease [1]. Introducing this pathogen into the skin of susceptible hosts, either via the bite of an infected tick or by injection of culture-grown bacteria, leads to their subsequent dissemination to several tissues, including heart, joint, and neural tissues [2]. These spirochetes are notable in their ability to persist for months to years within host tissues, with intermittent reemergence promoting the acute localized inflammatory lesions that characterize Lyme disease. While these persistent bacteria elicit strong innate and adaptive immune responses, their fastidious growth requirements have hindered *in vitro* analyses to determine which elements of host immunity are most important for controlling these infections [3–7]. 

Most studies to assess immune responses against *B. burgdorferi* are performed using a well-described murine model of Lyme disease. Mice are a natural reservoir for *B. burgdorferi*, and persistent bacteria within certain inbred strains are associated with similar tissues and produce inflammatory pathology consistent with that exhibited in human patients, though the severity of disease can vary widely between different inbred mouse strains [8–10]. Infection studies using inbred strains have allowed identification of specific immune mediators that affect host clearance, such as Toll-like receptor 2 (TLR2) [11, 12], MyD88 [13, 14], CD14 [15, 16], IL-10 [17, 18], the chemokine KC [19], and the production of antibodies against critical *B. burgdorferi* antigens [20–24]. 

Studies elucidating the basis of *B. burgdorferi* clearance have relied heavily on two parameters, namely, seroconversion to bacterial antigens and detection of bacterial DNA in host tissues. Production of high antibody titers against certain *B. burgdorferi* antigens, which have been further characterized using western blot analyses, can protect animals from both tick-mediated and syringe challenge with *B. burgdorferi* [9, 22, 25]. The specific effects of antibodies and other immune mediators on *B. burgdorferi* clearance have traditionally been measured qualitatively by culturing murine tissues in sterile BSK medium and determining whether resident spirochetes can grow from these cultures [26]. More recently, real-time PCR techniques have been developed that can accurately quantify even minute *B. burgdorferi* levels in murine target tissues [17, 27, 28], and similar methods have been used to compare the upregulation of targeted murine and bacterial gene products within infected tissues [18, 29, 30]. The refinement of these techniques have greatly improved the usefulness of the murine model of Lyme disease, particularly in identifying immune mediators that are effective in controlling these unique pathogens.

While both ELISA techniques, to measure antibody levels, and PCR analyses, to determine *B. burgdorferi* levels, are widely used to assess the development of Lyme disease in infected animals, questions have been raised regarding how accurately these techniques assess the infection status. *B. burgdorferi* are known to be highly immunogenic, largely due to the wide range of lipoproteins that are produced in response to different environmental cues [6, 31, 32]. These lipoproteins all possess a triacyl modification on their amino terminus [33] that not only activates many different host immune cells through interaction with TLR2 [11, 34–36] but also provides potent adjuvant activity that significantly enhances antibody responses to these lipoproteins [37, 38]. This raises the possibility that mice receiving a significant inoculum may produce substantial *B. burgdorferi*-specific antibodies that do not truly reflect a response to an active infection, but alternatively reflect an antibody response to an initial inoculum that was quickly cleared; in these cases, differences between active and subclinical infection would only be apparent by subsequent western blot analyses. A second issue is that *B. burgdorferi* can persist in many different tissues, but the precise extracellular or intracellular microenvironment in which they persist, as well as the immunoprivileged status of that niche, is still being defined [39–42]. It is plausible that bacterial products from killed bacteria, such as DNA, might escape timely or complete clearance from those tissues, and, thus, subsequent assessment could falsely indicate that viable *B. burgdorferi* were persisting in those tissues. To address these issues, we have injected mice with various doses of live and heat-killed bacteria to determine whether significant and characteristic differences in both antibody production, as assessed by ELISA analyses, and detection of *B. burgdorferi* DNA, by PCR, can accurately reflect whether the mice were actively infected or were only exposed to a threshold level of bacterial antigens.

## 2. Materials and Methods

### 2.1. Infection of Mice with Borrelia burgdorferi

C57BL/6NCr (B6) mice were obtained from the National Cancer Institute: Frederick Animal Production Program (Frederick, MD). Mice were housed in the Department of Lab Animal Resources at the University of Toledo Health Sciences Campus according to the National Institutes of Health guidelines for the care and use of laboratory animals. All protocols were reviewed and approved by the Institutional Animal Care and Usage Committee. 

The clonal N40 isolate [43] of *B. burgdorferi* was generously provided by Steve Barthold (University of California, Davis) as a passage two culture after isolation from the urinary bladder of a Rag-1^−/−^ mouse. For all infections, a passage 4 culture was grown in BSK-II medium supplemented with 6% rabbit serum (Sigma Chemical, St. Louis, Mo, USA) for 3–5 days at 33°C and directly enumerated using a Petroff-Hauser's chamber and dark field microscopy. B6 naïve mice were infected with the indicated numbers of viable or heat-killed *B. burgdorferi* in a 20 *μ*L volume by intradermal injection into a shaven back. These bacteria remain intact after heat killing (55°C for 1 hour) based on visual inspection and counting by dark field microscopy but are subsequently unable to grow in BSK medium (data not shown).

### 2.2. Immunoglobulin (Ig) Quantification

Serum was obtained at the indicated times by either retroorbital bleeding or exsanguination, and Ig content was assessed using previously described ELISA techniques [17]. Briefly, microtiter plates were coated with either sonicated *B. burgdorferi *or goat antibodies to mouse IgG, IgM, and IgA (Southern Biotech, Birmingham, Ala, USA). Multiple serum dilutions were added to plates for 90 min at 37°C, and bound murine Ig was detected by addition of isotype-specific HRP-conjugated antibodies (Southern Biotech). Ig content was quantified by comparison to standard curves constructed by using purified Ig of the appropriate isotype (Southern Biotech).

### 2.3. Western Blot Analysis

One hundred twenty *μ*g of sonicated cN40 isolate were electrophoresed in a 4–12% Bis-Tris gel (Invitrogen) containing a single large well, transferred to Immobilon-P membrane (Millipore, Bedford, Mass, USA), and immunoblotted using a Surf Blot apparatus (Idea Scientific Company, Minneapolis, Minn, USA). Immune sera used to blot the membrane were obtained from B6 mice at day 28 after inoculation with viable or heat-killed *B. burgdorferi*. Antibody-antigen complexes were detected by addition of HRP-conjugated antibodies specific for total murine Ig (Southern Biotech) and visualized by chemiluminescence. Multiple film exposure times were acquired for each blot to ensure that all protein bands were recorded irrespective of concentration variances between samples.

### 2.4. DNA Preparation

Murine skin tissues encompassing (6 mm diameter) the Bb injection site were harvested from experimental animals sacrificed at the indicated times after injection, and DNA was prepared from individual tissues as previously described [10]. Briefly, tissue specimens were incubated in 0.1% collagenase A (Roche Diagnostics, Indianapolis, Ind, USA) at 37°C overnight, followed by the addition of an equal volume of 0.2 mg/mL proteinase K (Invitrogen, Carlsbad, Calif, USA) and incubation overnight at 55°C. DNA was recovered by multiple phenol-chloroform extractions and ethanol precipitation and includes digestion of contaminating RNA in 1 mg/mL DNase-free RNase (Sigma), with the final sample resuspended in 500 *μ*L of TE buffer. The DNA content was quantified by absorbance at 260 nm, and working samples were diluted to 50 *μ*g/mL for quantitative real-time PCR analyses.

### 2.5. Quantification of B. burgdorferi in Mouse Tissues

The number of spirochetes resident in the different murine target tissues were determined via PCR analyses using a LightCycler (Roche Diagnostics) rapid fluorescence temperature cycler based on our previously described protocols [17, 28]. Briefly, amplification was performed on 100 ng of template DNA in a 10 *μ*L final volume containing 50 mM Tris (pH 8.3), 3 mM MgCl_2_, 4.5 *μ*g of bovine serum albumin, 200 *μ*M of each deoxynucleoside triphosphate, a 1 : 10,000 dilution of SYBR Green I (Molecular Probes, Eugene, Ore, USA), 1 *μ*M of each primer (Integrated DNA Technologies, Coralville, Iowa, USA), and 0.5 U of Platinum *Taq* DNA Polymerase (Invitrogen). Copy numbers for the mouse *nidogen* and *B. burgdorferi rec*A genes present in each sample were calculated by extrapolation to standard curves using LightCycler software (Roche Diagnostics). The reported data represents *rec*A values that were corrected by normalization based on the *nidogen* (*nid*) gene copy number. The oligonucleotide primers used to detect mouse *nidogen* were nido.F (5′-CCA GCC ACA GAA TAC CAT CC-3′) and nido.R (5′-GGA CAT ACT CTG CTG CCA TC-3′). The oligonucleotide primers used to detect *B. burgdorferi rec*A were nTM17.F (5′-GTC GAT CTA TTG TAT TAG ATG AGG CTC TCG-3′) and nTM17.R (5′-GCC  AAA  GTT  CTG  CAA  CAT  TAA CAC  CTA  AAG-3′).

### 2.6. Statistical Analyses

The statistical significance of the quantitative differences between the different sample groups was determined by application of Student's two-tailed *t*-test; *P* values that were ≤0.05 were considered significant.

## 3. Results

### 3.1. Quantification of Bb-Specific Ig Levels in Serum

**Figure 1:**
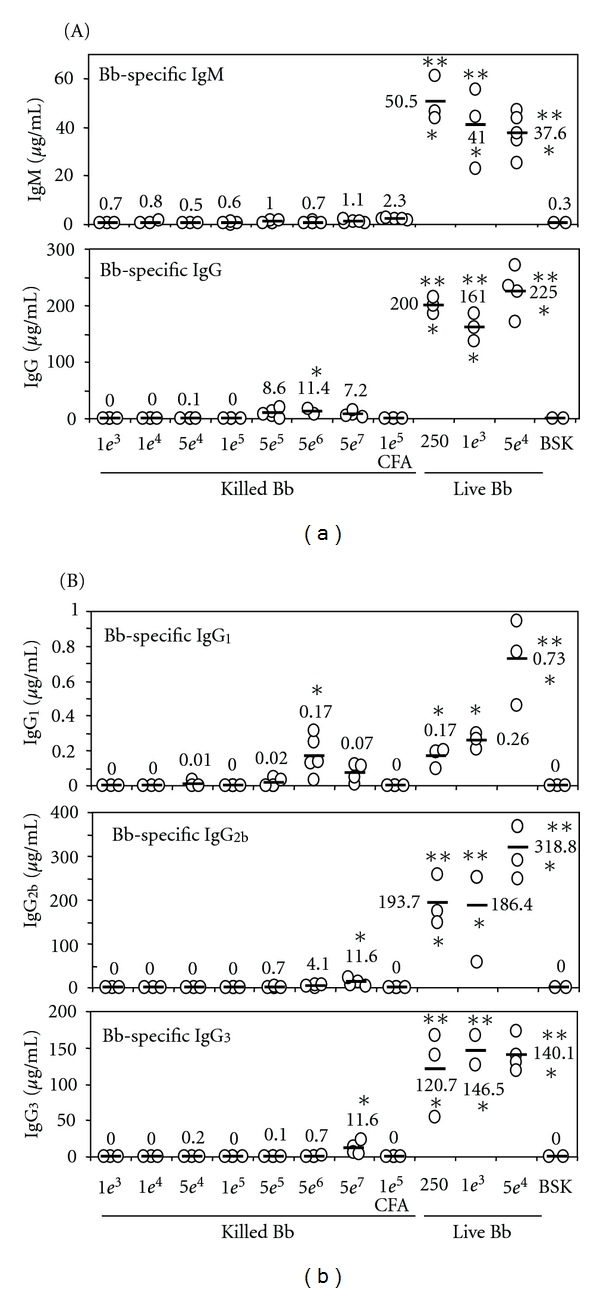
Comparison of Bb-specific antibody levels produced during active infection versus exposure to high numbers of killed bacteria. (a) Groups of B6 mice were injected with a single dose of live or heat-killed Bb, and serum was collected at either 2 or 4 weeks after infection; in some cases the killed Bb inoculum included an equal volume of complete Freund's adjuvant (CFA). The doses injected ranged from 1000 (“1*e* ^3^”) to 5 × 10^7^ (“5*e* ^7^”) killed bacteria, and 250 to 5 × 10^4^ (“5*e* ^4^”) live Bb. Individual sera were assessed for Bb-specific IgM content at 2 weeks after infection and for IgG content at 4 weeks after infection by ELISA analyses. Each circle represents the serum value for an individual animal, and the number beside the bar indicates the average value for that group. *indicates values that are significantly different from control mice (Un); **indicates values that are significantly different from mice injected with killed bacteria. (b) The sera assessed for IgG content in (a) were also assessed for the levels of the indicated individual IgG isotypes using similar ELISA techniques.

We initially wanted to determine whether distinct quantitative differences are detectable in antibody levels produced during an active Bb infection versus bacterial exposures that are quickly resolved. To address this, groups of B6 mice were injected with different doses of live or heat-killed bacteria, and the Bb-specific Ig content of individual sera collected either 2 or 4 weeks after infection was compared by ELISA analysis. Sera from control mice that were injected only with BSK II medium contained no Bb-specific IgG and minimal levels of Bb-specific IgM (Figure 1), which reflects the presence of natural Bb-specific IgM in naïve mice, as previously reported [44, 45]. Mice injected with live bacteria showed higher Bb-specific IgM levels at 2 weeks (Figure 1(a)) than at 4 weeks (data not shown), and while low levels of Bb-specific IgG were seen at 2 weeks after infection (data not shown), the levels were much higher at 4 weeks post-infection (Figures 1(a) and 1(b)). Injecting a single dose of heat-killed Bb into B6 mice, with or without CFA, did not elicit significantly enhanced IgM levels compared to mice receiving BSK II medium alone (Figure 1(a)), even at a dose of 5 × 10^7^ killed Bb. However, injection of as few as 250 live bacteria increased the Bb-specific IgM levels by over 200-fold compared to mice receiving the highest dose of killed bacteria (*P* ≤ 1.6 × 10^−5^) by 2 weeks post-infection. A somewhat similar trend was seen in IgG levels at 4 weeks post-infection, where a dose of 5 × 10^6^ killed Bb was required to significantly increase Bb-specific IgG levels compared to naïve mice (*P* = 0.011). Again, injection with as few as 250 live bacteria increased the Bb-specific IgG levels by over 20-fold compared to mice receiving the highest dose of killed bacteria (*P* ≤ 2.3 × 10^−6^) by 4 weeks post-infection. These data indicate that significant quantitative differences in Bb-specific antibodies are readily detected in sera from animals that sustain an active infection compared to those exposed to relatively high numbers of killed Bb that are quickly cleared. 

To determine whether these differences in IgG production extended to all IgG isotypes, similar ELISA analyses were performed and developed using isotype-specific detection antibodies (Figure 1(b)). The production of IgG_2b_ and IgG_3_ isotypes showed a similar trend as reported for Bb-specific IgG, in that significant levels of Bb-specific antibodies were only observed at the very highest dose of killed bacteria, while administration of as few as 250 live Bb resulted in ≥10-fold increase in Bb-specific antibodies (*P* = 0.001 and 0.01, resp.). In contrast, IgG_1_ production appeared much more responsive to killed bacteria, with significant increases in Bb-specific IgG_1_ elicited in response to 5 × 10^6^ killed bacteria, and these levels were similar to those produced in response to all live bacteria inocula, except for the highest dose. However, the overall quantities of IgG_1_ antibodies were ≥200-fold less than the other isotypes assessed, and thus represent a minor component of the total IgG response.

### 3.2. Comparison of Bb Antigens Recognized by Antisera to Live and Killed Bb

**Figure 2:**
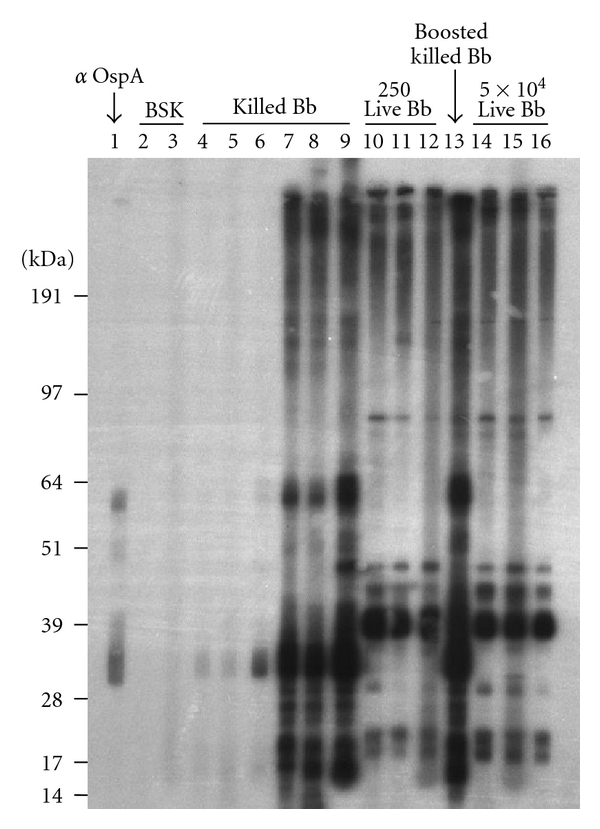
Antibodies produced against live and killed spirochetes recognize unique Bb antigens. Sera were collected from individual mice 4 weeks after injection with a single dose of either BSK II medium (lanes 2-3), 5 × 10^7^ killed Bb (lanes 4–9), 250 live Bb (lanes 10–12), or 5 × 10^4^ live Bb (lanes 14–16); one group initially received 10^5^ killed Bb + CFA, with a boost 3 weeks later with 10^5^ killed Bb + IFA, and sera collected 3 weeks later (lane 13). All sera were diluted either 1 : 25 (lanes 2-3 and 7–9) or 1 : 500 before using to immunoblot membranes containing electrophoresed Bb. A mAb specific for Bb OspA, H5332 [49], was included as a marker for OspA reactivity (lane 1).

Bb can rapidly modulate a large number of surface-exposed and other lipoproteins during their natural infection cycle from arthropod to vertebrate host [31, 32, 46–48]. Therefore, it is likely that the antigens that are immunologically recognized during an active infection differ from those in a killed Bb exposure that is rapidly cleared, and could be used to further confirm the significant differences in antibody production observed in the ELISA analyses. To address this, sera were collected from mice injected with various doses of live and killed Bb at 4 weeks post-infection, and used for western blot analyses to compare the range of Bb antigens that are recognized by each serum (Figure 2). Mice injected only with BSK II medium (lanes 2-3) possessed no detectable Bb-reactive antibodies, while a mAb specific for Bb OspA (lane 1) appeared to detect both the monomeric and dimeric forms of this lipoprotein. As expected based on the ELISA analyses (Figure 1), an approximately 20-fold higher concentration of serum from animals injected with killed bacteria was needed to effectively visualize Bb antigens compared to sera generated against live bacteria (Figure 2). Sera from mice that received either a single (lanes 7–9) or boosted dose (lane 13) of killed Bb demonstrated a protein recognition pattern that was distinct from mice receiving either a low (lanes 10–12) or high (lanes 14–16) dose of live Bb. These studies indicate that significant quantitative and qualitative differences are apparent in the antibodies produced between mice that undergo an active infection with Bb and those which rapidly clear a relative large bacterial inoculum.

### 3.3. Persistence of Bb DNA in Skin Tissues

**Figure 3:**
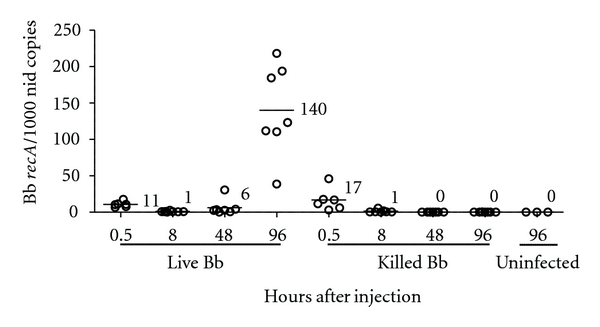
DNA from killed Bb is rapidly cleared from infection sites and is undetectable by PCR. Groups of mice were injected intradermally with 10^4^ of either live or heat-killed Bb. Mice were sacrificed at the indicated times after injection, and a 6 mm skin sample encompassing the injection site was excised for DNA preparation. The relative Bb levels were assessed by real-time PCR using *recA* primers and normalized relative to the murine *nid* content. Each circle represents Bb *recA* levels relative to 1000 *nid* copies for an individual animal, and the number beside the bar indicates the average value for that group. These data reflect the combined results of two separate experiments.

PCR-based assays are commonly used to quantify differences in Bb levels within host tissues for a number of model systems; however, it is not clear how long the DNA from killed bacteria might persist in those tissues and provide inaccurate estimation of the viable Bb numbers. To address this, parallel groups of mice were injected intradermally with either live or killed Bb, and skin tissues were harvested and assessed at different times after injection for the presence of bacterial DNA by real-time PCR using our described protocols [17, 28]. Skin tissues harvested immediately after injection of equal numbers of live or killed Bb showed the presence of similar numbers of Bb genomes by PCR analyses (Figure 3), and, by 8 h after injection, these numbers have substantially decreased to similar low levels in animals receiving both live and killed bacteria. After 8 h after injection, almost no Bb genomes were detected in skin tissues of animals receiving killed bacteria (Figure 3), and more extensive studies showed that no Bb genomes were detected in skin, ear, ankle, or heart tissues of mice receiving killed bacteria at 2 and 4 weeks after injection (data not shown). Alternatively, mice receiving live bacteria showed a gradual increase in skin Bb levels after 8 h following infection, with the highest bacterial numbers detected at 96 h after infection (Figure 3), and reduced but consistent levels noted at 2 and 4 weeks after injection (data not shown). These findings suggest that killed Bb are rapidly cleared from skin tissues and that their genome content can no longer be detected by PCR within hours of being killed.

## 4. Discussion

The spirochetal pathogen *B. burgdorferi* (Bb) is an obligate parasite that cycles efficiently between vertebrate and arthropod hosts and persists for extended periods within various host tissues. Antigenic variation has been described to play a putative role in immune evasion, but other mechanisms by which these bacteria evade clearance from immunocompetent hosts are not well defined. The fastidious growth requirements of these spirochetes make it difficult to design stringent *in vitro* analyses that accurately reflect host conditions. Therefore, infection studies using inbred mouse lines are the gold standard for addressing host-Bb interactions. Both ELISA and western blot analyses have been useful in measuring the critical antibody responses to Bb infection, including the identification of immunoreactive bacterial products that can confer protective immunity and in determining infection rates by seroconversion. Similarly, the recent development of quantitative real-time PCR techniques has allowed researchers to distinguish important differences in immune clearance between different murine model strains and, thus, identify mechanisms that are critical for clearance of these persistent bacteria. The sensitivity and specificity of these assays are well documented, but questions have arisen as to their abilities to differentiate between active infections versus those produced by residual and/or stimulatory bacterial products that persist subsequent to bacterial killing. Our study attempts to clarify the limitations of these techniques in assessing the murine infection model of Lyme disease.

*B. burgdorferi* is notable in that ≥8% of putative protein-coding genes contain a signal peptide “lipobox” region, suggesting that these gene products are recognized by lipid modification enzymes that produce triacylated lipoproteins [6, 7, 33]. These lipoproteins can be secreted across the cytoplasmic membrane to the outer membrane [50], where they are not only recognized by the adaptive immune responses but can also interact with TLR2 on a number of different immune cell types to induce inflammatory responses [11]. As a result of these interactions, Bb lipoproteins are highly immunogenic and possess endogenous adjuvant activities that make them attractive vaccine candidates [38, 51, 52], such as the OspA-based LYMErix vaccine [53]. Infection with as few as 20 organisms can lead to the production of high levels of Bb-specific antibodies by 2 to 4 weeks after infection, which corresponds with a subsequent decrease of bacterial numbers in host tissues, suggesting the importance of these antibodies in controlling bacterial numbers [22, 54, 55]. 

While increases in Bb-specific antibody levels are often used as an indicator of infection, it is possible that this correlation is misleading, since even a Bb inoculum that is rapidly cleared by the innate immune responses would result in the presentation of a substantial number of immunogenic lipoproteins to T and B cells, which could potentially elicit antibody levels that approach those produced by infected animals. However, we found that mice injected intradermally with a single dose of either live or heat-killed Bb produced sera with distinct differences. A dose of at least 5 × 10^6^ killed bacteria was required to elicit detectable Bb-specific antibodies, but even 5 × 10^7^ killed bacteria elicited 20- to 400-fold less Bb-specific antibody levels than any tested doses of live bacteria. These large differences were noted in all Ig isotypes tested except for IgG_1_ production. Overall, these data suggest that significant differences are apparent between the antibody levels produced in response to a productive Bb infection versus exposure to killed bacteria and that properly controlled ELISA analyses can reliably distinguish the two.

During their natural infection cycle, Bb must quickly adapt to a wide range of arthropod and vertebrate environments in order to survive. Many of these changes are associated with rapid expression changes involving multiple surface lipoproteins [56]. For example, OspA is highly expressed on the surface of Bb within the tick midgut or when cultured in BSK medium at ambient temperatures [47]. However, the process of tick feeding or increases in temperature cause the rapid downregulation of OspA and subsequent upregulation of numerous lipoproteins associated with mammalian infection, such as OspC [46, 57], the Erp family proteins [30], and different modifications of the *vlsE*-expressed lipoprotein [58]. Thus, Bb introduced into a murine host will differentially express a large number of different lipoproteins during the course of infection and should subsequently present a much broader range of antigens to host immune cells than Bb that are rapidly killed. Our studies determined that the Bb proteins recognized by sera from mice injected with killed Bb were distinct from the proteins recognized by sera from mice injected with live Bb. Based on size and the OspA control, the response to killed Bb appeared largely directed against protein bands consistent with OspA monomers (~31 kDa) and dimers (~62 kDa), while the response to live Bb appeared directed to proteins with band sizes consistent with p39 (~39 kDa), flagellin (~41 kDa), and p93 (~93 kDa); these different reactivities to proteins recognized on live Bb have been previously noted by different investigators [8, 46, 59–61]. Because these changes in antigen expression have been shown to correspond with active infection, our western blot data confirms that the significant differences in ELISA values can accurately distinguish between animals that experienced a progressive Bb infection versus an exposure to bacteria that could not adapt to escape host clearance. 

Subsequent to inoculation in a murine host, Bb are known to disseminate from the skin and persist in a wide range of tissues (including the skin). While the precise environment that these bacteria prefer to persist within is not well defined, they are believed to largely exist extracellularly. Bb can associate with collagen bundles [62, 63], decorin-associated tissues [41, 64], or relatively avascular spaces throughout their host [65], all of which might provide some protection from immune recognition/clearance. Alternatively, Bb might be able to persist within some immune cells subsequent to phagocytosis, with both live and killed bacteria appearing to remain intact for extended periods of time [66]. These possibilities suggest that even Bb that are killed within host tissues might persist for extended times and, thus, could allow their cellular contents to be detected by assays designed to detect viable persistent bacteria, such as PCR of infected tissues. We addressed this by injecting mice with live and killed Bb and following the Bb content in the skin over time by PCR analysis. Levels of PCR-detectable bacteria declined to similar low levels by 8 h after injection in both groups, likely due to both the degradation of DNA content from killed Bb, as well as the dissemination of live Bb away from the harvested skin injection site. However, the Bb DNA content in tissues receiving live bacteria increased to reach peak values by 96 h and subsequently remained low but significant. This pattern is consistent with previous reports of Bb persistence within skin tissues [8, 12]. Our current studies suggest that killed Bb and their cellular content are efficiently cleared in skin tissues and should not be detectable by PCR methodologies within hours of being killed. In summary, both serological and PCR-based methods of assessing Bb infection clearly distinguish between an established infection with live bacteria and exposure to even large numbers of bacteria that are cleared early by the innate responses.

##  Acknowledgments

This work was supported by the Scientist Development Grant 0335148N from the American Heart Association (R. M. Wooten) and, Public Health Service Grant R01-AI073452 from the National Institute of Allergy and Infectious Diseases (R. M. Wooten). The authors wish to thank Robert Blumenthal and Isabel Novella for helpful discussions in writing this paper.
